# RANDOMIZED COMPARATIVE CLINICAL TRIAL OF ARTEMISIA SIEBERI 5% LOTION AND CLOTRIMAZOLE 1% LOTION FOR THE TREATMENT OF PITYRIASIS VERSICOLOR

**DOI:** 10.4103/0019-5154.43209

**Published:** 2008

**Authors:** Farrokh Rad, Farzad Aala, Naser Reshadmanesh, Rokshana Yaghmaie

**Affiliations:** 1 *From the Department of Parasitology, Kurdistan University of Medical Sciences, Iran*; 2 *From the Department of Health, Kurdistan University of Medical Sciences, Iran*; 3 *From the Department of Dermatology, Kurdistan University of Medical Sciences, Iran*

**Keywords:** *Artemisia sieberi lotion*, *clotrimazole lotion*, *pityriasis versicolor*, *recurrence rate*

## Abstract

**Aims:**

To compare the therapeutic effects of topical Artremisia sieberi 5% lotion with topical clotrimazole 1% lotion in the treatment of pityriasis versicolor.

**Materials and Methods:**

100 patients with pityriasis versicolor and microscopic identification of Malassezia furfur were randomly assigned to treatment with either topical Artemisia sieberi 5% lotion (group 1) or topical clotrimazole 1% lotion (group 2) for 2 weeks. Group 1 and group 2 consisted of 51 and 49 patients respectively. The patients were evaluated both clinically and mycologically at baseline and every 2 weeks for a period of 4 weeks.

**Results:**

At the end of the second week, clinical cure rates were 86.3% and 65.3% for group 1 and group 2 respectively (*P*<0.01), but at the same time mycological cure rate was 92.2% for group 1 and 73.5% for group 2 (*P*<0.05). At the end of the fourth week, clinical cure rates were 86.3% and 59.2% for group 1 and group 2 respectively (*P*<0.01), and at the same time mycologic cure rate was 96.1% for group 1 and 65.3% for group 2 (*P*<0.01).

**Conclusions:**

The results of this study demonstrated that Artemisia sieberi 5% lotion was more effective than clotrimazole 1% lotion in the treatment of pityriasis versicolor.

## Introduction

**Aims:** The aim of this study was to compare the therapeutic effects of topical Artremisia sieberi 5% lotion with topical clotrimazole 1% lotion in the treatment of pityriasis versicolor.

Pityriasis versiclor (tinea versicolor) is a common superficial fungal infection of the stratum corneum caused by the fungus Malassezia furfur classically affecting young people around the pubertal time.^1^,^2^ The pale type of pityriasis versicolor is thought to be due to a chemical produced by Malassezia that diffuses down and impairs the function of the pigment cells in the underlying skin.^3^ There are numerous ways of treating pityriasis versicolor topically and systemically. However the recurrence rate is very high^4^ and different studies have shown different therapeutic results.^5^–^7^ However, relapse is very common and researchers are working to produce more effective drugs.^8^

Propylene glycol 50% in water, imidazole and the older antidandruff shampoos as well as ciclopiroxolamine and terbinafine are effective topically. Short-term oral treatments with ketoconazole, itraconazole or fluconazole are very effective and the risk for side-effects minimized with short treatment regimen.^4^ In a double-blind clinical trial therapeutic effect of Artimisia sieberi 5% lotion was compared with that of clotrimazole 1% lotion in the treatment of pityriasis versicolor.^9^ In the microscopic study, negative direct smears observed in Artimisia sieberi group and clotrimazole group were 87.1% and 69% in the second week, and 100% and 61.5% in the fourth week (2 weeks after cessation of therapy) respectively.^9^

Artimesia sieberi is a native shrub growing in northern Iran.^10^,^11^ Alpha- and beta-thujones are important herbal medicines and food additives.^12^ Alpha-thujone generally is considered to be the principal active ingredient of worm wood oil.^13^–^15^

LD50 of alpha-thujone in rats is 500 mg/kg by oral route and 5000 mg/kg in rabbits by way of dermal contact. At a concentration of 4% alpha-thujone was negative in dermal irritation and maximization tests in humans.^16^ Herbal medicines have been popular among the people and have been used in traditional medicine for treatment of different disorders. Therefore we decide to assess the effect of Artemesia sieberi in treatment of pityriasis versicolor. The aim of the present study was to compare efficacy of topical Artemisia sieberi 5% lotion with that of clotrimazole 1% lotion in the treatment of pityriasis versicolor.

## Materials and Methods

The study was a randomized, double-blind clinical trial. After informed consent, 102 patients with pityriasis versicolor were enrolled in the study. The diagnosis was made on clinical examination and confirmed with microscopic examination of scales soaked in 10-15% potassium hydroxide.

This study was approved by Ethical Review Committee of Kurdistan University of Medical Sciences and conformed to the ethical guidelines of the 1975 Declaration of Helsinki.

Inclusion criteria were as follows: clinical diagnosis of pityriasis versicolor and its confirmation with microscopic examination in patients between 15 and 45 years of age, and involvement of less than 10% of the total body surface by skin lesions.

Pregnant and lactating women were excluded from the study. The patients were randomly divided into two groups. Group 1 used 1-2 ml of Artimisia sieberi 5% topical lotion produced by Barij Essence Pharmaceutical Company in Iran, twice daily and group 2 applied 1-2 ml of clotrimazole topical lotion on the lesions twice daily, for two weeks.

Of 102 patients, 100 completed the treatment course (51 patients in group 1 and 49 patients in group 2 respectively). Two patients were lost to follow-up and excluded from the study. Disappearance of all skin lesions on inspection was our criterion for clinical cure and negative direct smear in microscopic study was regarded as mycological cure.^17^

Skin assessment and microscopic examination of skin smears were performed at baseline, after completion of the two-week treatment course, and two weeks after cessation of the topical agents. The phone numbers and addresses of the patients were recorded in their files. One of our colleagues was responsible to call the patients to remind them of the follow-up visits, but in the event they did not come, he referred to them to take skin smear and evaluate the patients for clinical cure rate (assessment of the clinical cure rate for all of the patients was performed by the same person). Results of the clinical and microscopic examinations and demographic data of the patients were recorded in a check- list for each of the patients.

The data were processed with SPSS Win13 software and data analysis was performed by means of independent *t* test, Chi- square tests, and Fisher's exact test.

## Results

Analysis of the two treatment groups at baseline, as explained in the following paragraphs revealed no significant differences between the group 1 and group 2 patients in relation to age, sex distribution, number of weekly baths, distribution and location of the skin lesions, occupation, disease duration, and family history of the patients; suggesting that the randomized arms were equivalent.

There were 27 (27%) female and 73 (73%) male patients; sex distribution was similar in both groups (70.6% male in group 1 and 75.5% male in group 2) (*P* = 0.65).

The mean age of the patients was 26.5 ± 9.8 (range 15-45 years) (*P* = 0.26). The mean numbers of weekly baths were 1.5 ± 0.2 and 1.8 ± 0.25 for group 1 and group 2 respectively. The mean values for disease duration in group 1 and group 2 were 3.09 ± 0.43 and 3.61 ± 0.51 months respectively.

From the occupational point of view, no significant difference was noted between the two groups (*P* = 0.31). Distributions of lesions over different parts of the body (Neck, chest, abdomen, back arms…) was similar in both groups (*P* = 0.67).

19.6% of the patients of group 1 and 16.3% of the patients of group 2 had positive family history of pityriasis versicolor (*P* = 0.76).

Clinical assessment of the patients, after two weeks revealed that 86.3% of the patients of group 1 and 65.3% of the patients of group 2 achieved success, which was statistically significant (*P* < 0.05). Mycological examination of the skin smears at the same time demonstrated cure rates of 92.2% and 73.5% for group 1 and group 2 respectively, which showed a significant difference (*P* < 0.05).

After four weeks (2 weeks after cessation of the treatments) clinical cure rates were 86.3% and 59.2% (*P* < 0.01) and mycological cure rates at the same time were 96.1% and 65.3% (*P* < 0.01) for group 1 and group 2 respectively (Table 1).

**Table 1:** Comparison of the therapeutic effects of Artemisia sieberi lotion with clotrimazole lotion based on clinical examination and skin smear after 2 and 4 weeks of the treatment regimens

Group	Improvement N (%)	Lack of improvement N (%)	X^2^	*P*
Clinical examination				
2 weeks				
Artemesia	44 (86.3)	7 (13.7)	6.1	0.01
Clotrimazol	32 (65.3)	17 (34.7)		
4 weeks				
Artemesia	44 (86.3)	7 (13.7)	9.3	0.002
Clotrimazol	29 (59.2)	20 (40.8)		
Skin smear				
2 weeks				
Artemesia	47 (92.2)	4 (7.8)	6.43	0.01
Clotrimazol	36 (13)	13 (26.5)		
4 weeks				
Artemesia	49 (96.1)	2 (3.9)	15.3	0.000
Clotrimazol	32 (65.3)	17 (34.7)		

Among the cured patients, the recurrence rates clinically (assessed only by inspection of the skin) and mycologically (assessed by microscopic examination of the skin smears) were 9.4% and 11.1% respectively, in the patients of group 2. However we noticed no recurrence of the skin lesions, clinically and mycologically in the patients of the group 1, which was statistically significant (*P* < 0.01).

## Discussion

At the end of the second week of this study, clinical assessment demonstrated a higher cure rate in Artemisia sieberi group than clotrimazole group (*P* < 0.05). At the same time, in microscopic study, rate of negative direct smears was higher in group 1 (*P*< 0.05).

After four weeks from the start of the treatment regimens clinical re-evaluation of the patients revealed a higher cure rate in group 1 than group 2 (*P* < 0.01). At the same time a higher number of the patients of group 1 had negative direct smears, compared with group 2 patients (*P* < 0.01). In this study the results of clinical evaluation and microscopic examination showed that Artemisia sieberi lotion was superior to clotrimazole lotion in the treatment of pityriasis versicolor. It is likely that active antifungal constituent(s) of Artemisia sieberi persisted in the superficial layer of skin for a longer period than did clotrimazole and prevented early recurrence of pityriasis versicolor.

In a similar double-blind clinical trial, by Mansouri and her colleague's significant clinical improvement, after two weeks was observed in 77.4% and 60.7%, and after 4 weeks in 93.5% and 57.1% of the patients in Artemisia sieberi group and clotrimazole group respectively. After two weeks Mansouri found negative direct smears in 87.1% and 69%, and after four weeks in 100% and 61.5% of the patients in Artemisia group and clotrimazole group respectively.^9^ In the above mentioned study the results of clinical assessment and microscopic examination were compatible with those of our study.

Clinical cure rates after two and four weeks were lower than mycological cure rates at the same time periods. Therefore accuracy of the laboratory- based care rates was higher than that of clinically- assessed cure rates.

Occasionally in the pale type of pityriasis versicolor white marks are permanent for unknown reasons, persisting long after the scaling and yeasts have gone and despite exposure to the sun.^3^

In such cases results of the direct smear of the hypopigmented lesions are negative and clinically these patients may be misdiagnosed as cases of untreated pityriasis versicolor. Therefore sometimes clinical assessment and laboratory results may be incompatible. It is very likely that antifungal effect of Artemisia sieberi lotion is due to alpha- thujone.

The mechanism by which alpha- thujone is of therapeutic benefit in pityriasis versicolor is unclear at present. Alpha- thujon is a constituent of essential oils derived from a variety of plants including Artemisia species, sage and the Thuja tree.^14^,^16^ Alpha- thujone has also antihelminthic, insecticidal, and antinociceptive properties.^14^,^18^,^19^

## Conclusion

This double- blind clinical trial shows that Atremisia sieberi 5% lotion is more effective than clotrimazole 1% lotion in the treatment of pityriasis versicolor. According to the results of our study, recurrence rate is expected to be much lower with Artemisia sieberi 5% lotion than clotrimazole 1% lotion after cessation of the therapeutic agents. This study emphasizes the need for definitive studies to address this issue in the future.
